# Characterizing the urobiome in geriatric males with chronic indwelling urinary catheters: an exploratory longitudinal study

**DOI:** 10.1128/spectrum.00941-24

**Published:** 2024-10-10

**Authors:** Emma Stewart, Baylie R. Hochstedler-Kramer, Mark Khemmani, Nina M. Clark, Jorge P. Parada, Ahmer Farooq, Chirag Doshi, Alan J. Wolfe, Fritzie S. Albarillo

**Affiliations:** 1 Department of Microbiology and Immunology, Stritch School of Medicine, Loyola University Chicago, Maywood, Illinois, USA; 2 Division of Infectious Diseases, Loyola University Medical Center, Maywood, Illinois, USA; 3 Infectious Disease and Immunology Research Institute, Loyola University Chicago, Maywood, Illinois, USA; 4 Department of Urology, Loyola University Medical Center, Maywood, Illinois, USA; niversity of Arkansas for Medical Sciences, Little Rock, Arkansas, USA

**Keywords:** urinary tract infection, urine microbiome, catheter-associated urinary tract infection, microbiome, urobiome

## Abstract

The impact of chronic indwelling urinary catheters (IUCs) on the composition and stability of the urinary microbiota remains unknown. The primary aim of this study was to describe the urinary microbiomes of geriatric males with chronic IUCs. A secondary aim was to explore clinical catheter-associated urinary tract infection (CAUTI) courses of the participants. Geriatric male patients with chronic IUCs were followed longitudinally. Catheterized urine, catheter tips, and both urethral and periurethral swabs were collected from participants at monthly intervals. Microbes were isolated and identified from each specimen using an enhanced culture method called expanded quantitative urine culture (EQUC) and targeted 16S rRNA gene DNA sequencing. Microbial outcomes were examined both in the absence of urinary symptoms and in the context of clinical diagnosis of CAUTI. Ten male participants (mean age 86 years) were enrolled. Urinary microbiomes differed for each participant. However, within each individual, microbiomes were similar over time and across niches (bladder, catheter, urethra, and periurethra). Within-niche microbiomes differed across individuals, and this was observed over time. The most abundant bacteria isolated from all niches were known uropathogens. Six of 10 individuals met diagnostic criteria for CAUTI at least once during the 12-month observation period, but no evidence of this or antibiotic treatment/response was discernable in our monthly samples. The microbiomes of each participant were unique and remained similar over time and across niches. Longitudinal EQUC or 16S rRNA gene sequencing data could be useful to clinicians when diagnosing or treating possible CAUTI.

**IMPORTANCE:**

Catheter-associated urinary tract infections (CAUTIs) are serious but preventable nosocomial infections. The most common risk factor for developing CAUTI is prolonged use of indwelling urinary catheters (IUCs). This study provides the first longitudinal description of the urinary microbiomes of geriatric males with chronic IUCs, in the absence of urinary signs and symptoms, as a first step toward enhancing our knowledge of the impact of chronic IUCs on the composition and stability of the urinary microbiota. This is an understudied area, particularly for males.

## INTRODUCTION

Catheter-associated urinary tract infections (CAUTIs) are serious but preventable nosocomial infections that are responsible for over 13,000 deaths annually; life-threatening comorbidities, such as blood stream infections; pyelonephritis; increased length of hospital stays; and increasing rates of antimicrobial resistance (1–3). Two decades ago, CAUTIs accounted for nearly 34% of all healthcare-acquired infections in the United States, but the incidence has since been declining with widespread adoption of prevention measures (3, 4).

The most common risk factor for developing CAUTI is prolonged use of urinary catheters (5).The risk of CAUTI increases 3%–7% each day an indwelling urinary catheter (IUC) remains inserted, with chronic IUC use associated with a CAUTI risk of almost 100% (6, 7). While up to 20% of hospitalized patients are catheterized short term during inpatient admissions, the overall prevalence of chronic catheterization in the United States is not known. However, it is estimated that 5% of long-term care facility residents have chronic catheters in place (8). If the patient is not a candidate for intermittent catheterization, use of a chronic IUC is indicated for urinary retention and urinary incontinence (9).

The diagnosis of CAUTI requires the following three criteria be met: 1) the patient must have an IUC for 48 hours or more or have had an IUC removed in the last 48 hours; 2) the patient must have either a fever of at least 38°C, suprapubic tenderness, costovertebral angle tenderness, urinary urgency, urinary frequency, or dysuria; and 3) the patient’s standard urine culture (SUC) must grow no more than two microbial species with at least one species present with at least 10^5^ colony-forming units (CFUs)/mL (2). Due to the growing threat of antimicrobial resistance, clinical practice guidelines do not recommend treating asymptomatic bacteriuria in patients with IUCs; however, the practice remains a common clinical occurrence (10, 11). CAUTI isolates from ICU patients increasingly demonstrate antimicrobial resistance, with one study reporting that 48.2% of *Klebsiella* species causing CAUTI in long-term acute care hospitals are resistant to extended-spectrum beta-lactams (ESBL), with 23% resistant to carbapenems (12).

It has long been believed that a major mechanism of CAUTI pathogenesis is extraluminal introduction of bacteria into the urinary tract, with the catheter acting as a conduit for microbes to ascend to the bladder from the urethral meatus along the outside surface of the catheter (13). Other mechanisms include biofilm production, impaired host defenses secondary to catheter-induced inflammation, intraluminal stasis due to drainage failure, and intraluminal entry—when microbes gain entry to the lumen of the catheter due to contamination or bag drainage failure (3, 13, 14). However, the external bacteria etiology theory, as well as the diagnosis criteria for urinary tract infections (UTIs), was developed prior to the discovery that the urinary bladder is not sterile.

We now know that microbial communities inhabit the urinary tract, and predisposition to or protection against UTI risk is related to the composition of the bladder microbiota (also known as the urobiome) (15–20). With the use of 16S rRNA gene sequencing and expanded quantitative urine culture (EQUC) techniques, communities of live bacteria have been detected in urine specimens that were negative on SUC (11, 21, 22). The EQUC technique differs from SUC, in that it requires larger urine volumes and longer incubation times to grow microbes on a diverse set of medias in multiple atmospheric conditions. Recent evidence suggests that SUC is limited in its ability to detect uropathogens compared to EQUC; while SUC preferentially detects certain known microbes, such as *Escherichia coli*, EQUC is capable of detecting more clinically relevant uropathogens (23). Indeed, EQUC and 16S rRNA gene sequencing have provided growing evidence supporting the presence of distinct emerging uropathogens that grow poorly on SUC and are thus underreported and understudied as causes of UTI (24).

The existence of the urobiome thus calls into question two prevailing dogmas regarding CAUTI: (1) the diagnosis relies on SUC, which operates under the assumption that a state of bacteriuria is abnormal, and (2) the existing catheter-as-conduit pathogenesis theory assumes that uropathogens are introduced extraluminally from the external environment along the catheter–urethral interface. While the microbial profiles of catheter biofilms have been extensively studied, the urobiomes of patients with chronic IUCs have only been described in several studies (25–29). The impact of chronic IUCs on the composition and stability of the urinary microbiota remains unknown.

This longitudinal, exploratory study aims to describe the urinary microbiomes of a small sample of geriatric males with chronic IUCs. It also explores clinical CAUTI courses of the participants with available EQUC and 16S rRNA gene sequencing data with the intent to begin to determine how future studies can be designed to estimate the associations between CAUTI signs and symptoms and the urobiome in males.

## MATERIALS AND METHODS

### Recruitment and demographics

Following institutional review board (IRB) approval (IRB No. LU212677), 10 adult males, 18 years or older, were asked for consent and enrolled in a longitudinal, observational study. All participants had chronic IUCs requiring routine outpatient urethral catheter exchange. Patients were excluded from the study if they had antibiotic exposure for any reason in the last 30 days or since their last catheter exchange, a history of bladder augmentation or urinary diversion, active gross hematuria, a current CAUTI, signs and symptoms of sepsis, or concern for pyelonephritis. Demographic, past medical and surgical history, medication use data, and urine culture laboratory data were extracted from participants’ electronic medical records. A second protocol (IRB No. LU 217801) was obtained to extract additional data from medical records, including indication for chronic IUC, length of time of IUC in place, dates of clinical urine cultures, and response to antibiotic treatment.

### Specimen collection

Participants provided four specimens on the day of enrollment in the following order: catheter, periurethral swab, urethral swab, and catheterized urine. The IUC present prior to enrollment was removed, and the catheter was collected in a sterile collection cup. Swabs (BD Eswab, Fisher Scientific) from the periurethra and urethra (fossa navicularis) were obtained. A new IUC was inserted using a sterile technique following betadine cleansing of the urethral meatus. An initial catheterized urine specimen was obtained (BD Vacutainer, Fisher Scientific) following catheter placement. The removed IUC (hereafter referred to as “catheter”), periurethral swab and urethral swab (hereafter referred to as “periurethra” and “urethra,” respectively), and catheterized urine (hereafter referred to as “bladder”) were held at 4°C for less than 4 hours before delivery to the research team for processing and analysis. Upon receipt, specimens were first processed with EQUC, as described below. The remaining specimen solutions were preserved with a nucleic acid preservative (10% AssayAssure, Sierra Molecular) (30) and stored at −80°C.

Following enrollment, patients presented to the outpatient urology clinic once monthly for catheter exchange. At each monthly visit, catheterized urine specimens were obtained prior to IUC removal by aspirating urine via the sterile channel from catheter tubing. The removed IUC, urethral swab, and periurethral swab were obtained at each visit and handled as described above. Patient history regarding CAUTI symptoms and antibiotic use since last visit was also obtained and documented.

Collected specimens from the initial day of enrollment are hereafter referred to as specimens associated with “time point (TP) 1.” The specimens collected at the subsequent monthly catheter exchange visits are hereafter referred to as specimens associated with “TP 2,” “TP 3,” “TP 4,” and so on.

### Clinical data collection

In addition to the data and specimen collection outlined above, a nurse coordinator called participants weekly to screen for signs or symptoms of CAUTI. CAUTI signs or symptoms were defined as the presence of at least one of the following: change in urine color, foul-smelling urine, urinary retention, abdominal pain or bladder pain, flank pain, and fever. If an SUC was ordered for a patient during the course of the study, the SUC results and data regarding antibiotic use, such as name of antibiotics, duration, and response to treatment, were obtained from the electronic medical records. While change in urine color or foul-smelling urine are not true signs of CAUTI (31), these signs are commonly assessed and documented as they contribute to providers’ clinical judgements. The diagnosis and treatment of CAUTI was deferred to the participant’s provider at each visit.

### Specimen processing

The proximal 0.5 cm of the urinary catheter was cut from the remaining catheter tubing with a sterile scalpel. The cut catheter tip was added to a sterilized specimen bag with 3 mL sterile phosphate-buffered saline and then sonicated for 5 minutes. The liquid solution was then processed with the bladder, urethral, and periurethral specimens in the following ways.

All specimens were cultured with EQUC protocols, as previously described (32). Briefly, 0.1 mL urine or 0.01 mL catheter, urethral, or periurethral buffered solutions were plated onto diverse types of media with incubation in diverse environments at 35°C for 48 hours. The detection level was set at 10 CFUs/mL for catheterized urine or 100 CFUs/mL for the other specimens, represented by 1 colony of growth on any plate. Following EQUC, morphologically distinct colonies were isolated and underwent matrix-assisted laser desorption/ionization time-of flight (MALDI-TOF) mass spectrometry for taxa identification. MALDI Biotyper 3.0 Realtime Classification software was used to analyze bacterial isolates; log score criteria were used to identify taxa at the species level, as previously described (23).

All specimens were also subjected to 16S rRNA gene sequencing. Genomic DNA was extracted from urine, catheter, and swab suspensions as follows. One milliliter of the urine or catheter suspension or 300 uL of the swab specimens was transferred to deepwell plates. Plates were centrifuged for 10 minutes at 1,300 x *g,* and all but 100 uL of the supernatant was removed. Enzymatic treatment consisting of 4 mg lysozyme, 3kU mutanolysin, 4 g lysostaphin, and 200U achromopeptidase in buffer (20 mM Tris, 2 mM EDTA, 1.2% Triton, pH 8.0) was added to each specimen and incubated at 37°C, 5 hours, shaking intermittently at 1,000 rpm (Eppendorf Thermomixer with SmartBlock DWP1000). Specimens were then processed using the DNeasy 96 Blood & Tissue kit as per the manufacturer’s instructions. To assess potential DNA contamination, an extraction negative control with no urine was processed with all specimens and sequenced in every run. To ensure reproducibility, each low biomass specimen of catheterized urine was extracted and sequenced in duplicate, whenever possible.

Specimens were sent for 16S rRNA gene sequencing at SeqCenter (Pittsburgh, PA). Specimens were prepared using the Zymo Research’s Quick-16S kit with phased primers targeting the V4 regions of the 16S gene (GTGYCAGCMGCCGCGGTAA and GGACTACNVGGGTWTCTAAT). Following clean up and normalization, specimens were sequenced on a P1-600cyc NextSeq2000 Flowcell to generate 2 × 301 bp PE reads. Adapters were trimmed. High-quality trimmed reads were obtained after low-quality reads were removed using Cutadapt. DADA2 software was used to process reads, including filtering, dereplication, chimera removal steps, and construction of an amplicon sequence variant (ASV) abundance table. The ASVs were annotated, using Bayesian LCA-based taxonomic classification method (33), which uses the NCBI database to achieve taxon identification.

ASVs with taxa confidence scores less than 70% were assigned “unclassified/unknown,” with one exception: when an ASV was assigned to the family Enterobacteriaceae, we used a lower confidence score, assigning that ASV to the genera *Escherichia* or *Klebsiella* because we have ample evidence that these assignments are valid from previous studies where we subjected similar samples to both 16S rRNA gene sequencing and an enhanced culture method called expanded quantitative urine culture. ASVs associated with the genus *Lysobacter* were removed as it is a known contaminant of the enzyme achromopeptidase included in the DNA extraction procedure. Low abundance ASVs were removed. Then, contaminant ASVs were determined via a scoring method that encompasses results from Decontam (34), nonparametric statistical testing (Kruskal–Wallis *P* < 0.5), mean comparison (i.e., mean counts of samples were less than mean counts of extraction controls), and whether sample read counts were less than 5 x greater than extraction controls.

### Data analysis

Within-specimen (alpha) diversity indices were computed using statistical packages phyloseq and vegan in R (R Foundation for Statistical Computing, Vienna, Austria) (35, 36). Observed (or richness) refers to the number of taxa per specimen, whereas evenness refers to the distribution of taxa within a specimen. The Shannon, Simpson, and inverse Simpson indices combine richness, evenness, and/or abundance. Tukey’s multiple comparison test was used to compare the alpha indices between niches with the *P*-value set to 0.05. We used Bray–Curtis distances to calculate between-specimen (beta) diversity. Beta diversity indicates species composition variation among the specimens. Bray–Curtis dissimilarity scores were calculated using the ecodist statistical package (37). Fisher’s exact test was used to analyze significance of the Bray–Curtis dissimilarity scores with a reference of 0.5. Jensen–Shannon Divergence (JSD) statistic was used to quantify composition stability over time; JSD values were calculated in RStudio. JSD values were calculated by comparing the microbiota of each niche at one time point with every other time point. The JSD axis matrix clusters time points by composition similarity, where more clustered points represent more similar microbiomes and further apart points represent more dissimilar microbiomes.

## RESULTS

### Demographics

A total of 10 geriatric males with chronic IUCs were enrolled. The cohort was predominantly Caucasian (90%) with a mean age of 85.6 years (range 73–96). Mean chronic IUC dwell time was 24.84 months (range 2.25–100.25) prior to enrollment. The indication for 90% of participants’ chronic IUC was urinary retention, primarily due to benign prostatic hyperplasia. One participant was catheterized due to urinary frequency and incontinence (Table 1).

**TABLE 1:** Demographics and patient characteristics

Characteristic	Value
Mean age (range)	85.6 (73–96)
Race (%)	
Caucasian	9 (90)
African–American	1 (10)
Ethnicity (%)	
Non-Hispanic or Latino	10 (100)
Hispanic or Latino	0 (0)
Mean months with chronic IUC prior to study enrollment (range)	24.84 (2.25–100.25)
No. patients with past urinary tract surgical history (%)	1 (10)
Indication for chronic IUC (%)	
Urinary retention due to BPH	5 (50)
Urinary retention due to neurogenic bladder	2 (20)
Urinary retention due to prostate malignancy	1 (10)
Urinary retention due to unknown	1 (10)
Urinary frequency and incontinence	1 (10)

Complete specimen sets were collected for four (40%) participants (participants 1, 2, 8, and 10); however, participant 10 only had five total sets of specimens processed. A complete list of number of study visits, sets of specimens collected, and sets of specimens processed for each participant is detailed in Table S1. Two participants were lost to follow-up, one was withdrawn due to chronic skilled nursing facility residence, and two died.

### Signs, symptoms, and CAUTI diagnoses

At 97.1% of the monthly follow-up examinations, the urinary catheter was exchanged. Of 305 total weekly phone call check-ins with the 10 participants, no urinary signs or symptoms were reported during 95.7% of calls. The few symptoms reported included change in urine color (3.3% of calls), foul-smelling urine (1.3%), urinary retention (0.7%), abdominal or bladder pain (0.7%), flank pain (0.3%), and fever (0.3%).

Six (60%) participants were diagnosed with CAUTI and treated with antibiotics at one or more time points during the study for a total of 15 total instances (participants 1, 2, 6, 7, 8, and 10). Of the 15 instances in which CAUTI was diagnosed and antibiotics prescribed, nine (60%) of the instances were not accompanied by recorded signs and symptoms of CAUTI, but microbes were isolated from SUC.

The clinical courses of the four participants for which specimens were collected at more than five time points are shown in Table 2. All four participants had SUC performed at least once during their monthly follow-up examinations. For three participants, SUC grew greater than 100,000 colonies/mL of a particular species; the participants were then prescribed antibiotics despite an absence of accompanying urinary signs or symptoms.

**TABLE 2:** Clinical course of participants 1, 2, 8, and 10^*a*^

	TP 1	TP 2	TP 3	TP 4	TP 5	TP 6	TP 7	TP 8
Participant 1								
Species present in bladder on EQUC	*Proteus mirabilis*	*Proteus mirabilis* *Klebsiella oxytoca* *Enterococcus faecalis*	*Proteus mirabilis* *Klebsiella oxytoca* *Unknown*	*Klebsiella oxytoca* *Unknown* *Proteus mirabilis* *Enterococcus faecalis*	*Proteus mirabilis* *Unknown*	*Klebsiella oxytoca* *Proteus mirabilis* *Unknown* *Enterococcus faecalis*	*Proteus mirabilis* *Klebsiella oxytoca* *Enterococcus faecalis*	
SUC result	Not performed	Not performed	Not performed	Not performed	Not performed	> 100 k colonies/mL *K. oxytoca* >100 k colonies/mL *P. mirabilis*	Not performed	
UTI signs or symptoms	None	None	None	None	None	None	None	
Antibiotic prescribed	None	None	None	None	None	Trimethoprim-sulfamethoxazole – both organisms susceptible	None	
Participant 2								
Species present in bladder on EQUC	*Enterococcus faecalis* *Serratia marcescens* *Pseudomonas aeruginosa* *Staphylococcus epidermidis*	*Serratia marcescens* *Pseudomonas aeruginosa* *Staphylococcus aureus*	*Serratia marcescens* *Citrobacter freundii* *Enterococcus faecalis* *Klebsiella pneumoniae* *Pseudomonas aeruginosa*	*Unknown* *Citrobacter freundii* *Klebsiella pneumoniae* *Enterococcus faecalis*	*Klebsiella pneumoniae* *Enterococcus faecalis* *Veillonella atypica* *Staphylococcus epidermidis*	*Enterococcus faecalis* *Klebsiella pneumoniae* *Serratia marcescens* *Pseudomonas aeruginosa*	*Klebsiella pneumoniae* *Enterococcus faecalis*	*Klebsiella pneumoniae* *Staphylococcus epidermidis* *Enterococcus faecalis* *Citrobacter freundii*
SUC result	Not performed	Not performed	Not performed	>100 k colonies/mL *K. pneumoniae*	Not performed	Not performed	Not performed	Not performed
UTI signs or symptoms	None	None	None	None	None	None	None	None
Antibiotic prescribed	None	None	None	Cephalexin – susceptible to cefazolin	None	None	None	None
Participant 8								
Species present in bladder on EQUC	*Providencia stuartii* *Unknown* *Klebsiella oxytoca* *Enterococcus faecalis* *Pseudomonas aeruginosa* *Proteus mirabilis*	*Alcaligenes faecalis* *Providencia stuartii* *Unknown* *Enterococcus faecalis* *Pseudomonas aeruginosa* *Klebsiella oxytoca*	*Enterococcus faecalis* *Klebsiella oxytoca* *Pseudomonas* *aeruginosa* *Staphylococcus epidermidis*	*Enterococcus faecalis* *Dermabacter hominis* *Candida albicans*	*Staphylococcus aureus* *Raoultella ornithinolytica* *Citrobacter braakii* *Enterococcus faecalis* *Citrobacter freundii*	*Citrobacter freundii* *Staphylococcus aureus* *Enterococcus faecalis* *Pseudomonas aeruginosa* *Unknown* *Klebsiella oxytoca*	*Serratia marcescens* *Citrobacter freundii* *Pseudomonas aeruginosa* *Staphylococcus aureus* *Enterococcus faecalis*	
SUC result	Not performed	Not performed	Not performed	Contaminated: three or more Gram-positive and Gram-negative organisms present	Contaminated: three or more Gram-positive and Gram-negative organisms present	Not performed	>100 k colonies/mL *Enterococcus* species	
UTI signs or symptoms	None	None	None	None	None	None	None	
Antibiotic prescribed	None	None	None	Ceftriaxone, then trimethoprim-sulfamethoxazole – no susceptibility testing performed	Ceftriaxone, then ciprofloxacin – no susceptibility testing performed	None	Ampicillin, then amoxicillin – no susceptibility testing performed	
Participant 10								
Species present in bladder on EQUC	*Enterococcus faecalis* *Klebsiella oxytoca* *Klebsiella pneumoniae*	*Klebsiella oxytoca* *Enterococcus faecalis* *Unknown*	*Unknown* *Enterococcus faecalis* *Klebsiella oxytoca*	*Klebsiella oxytoca* *Enterococcus faecalis*	*Klebsiella oxytoca* *Enterococcus faecalis*	Specimen not processed		
SUC result	Not performed	Not performed	Not performed	Not performed	Not performed	Contaminated: three or more Gram-positive and Gram-negative organisms present		
UTI signs or symptoms	None	None	None	None	None	Yes, change in urine color		
Antibiotic prescribed	None	None	None	None	None	Cephalexin – no susceptibility testing performed		

^*a*^ Most predominant species identified in the bladder using EQUC compared with SUC results, urinary signs or symptoms, and prescribed antibiotics with available susceptibility data at each time point. Species present in the bladder on EQUC listed in order of abundance.

### Microbiota composition

**Fig 1:**
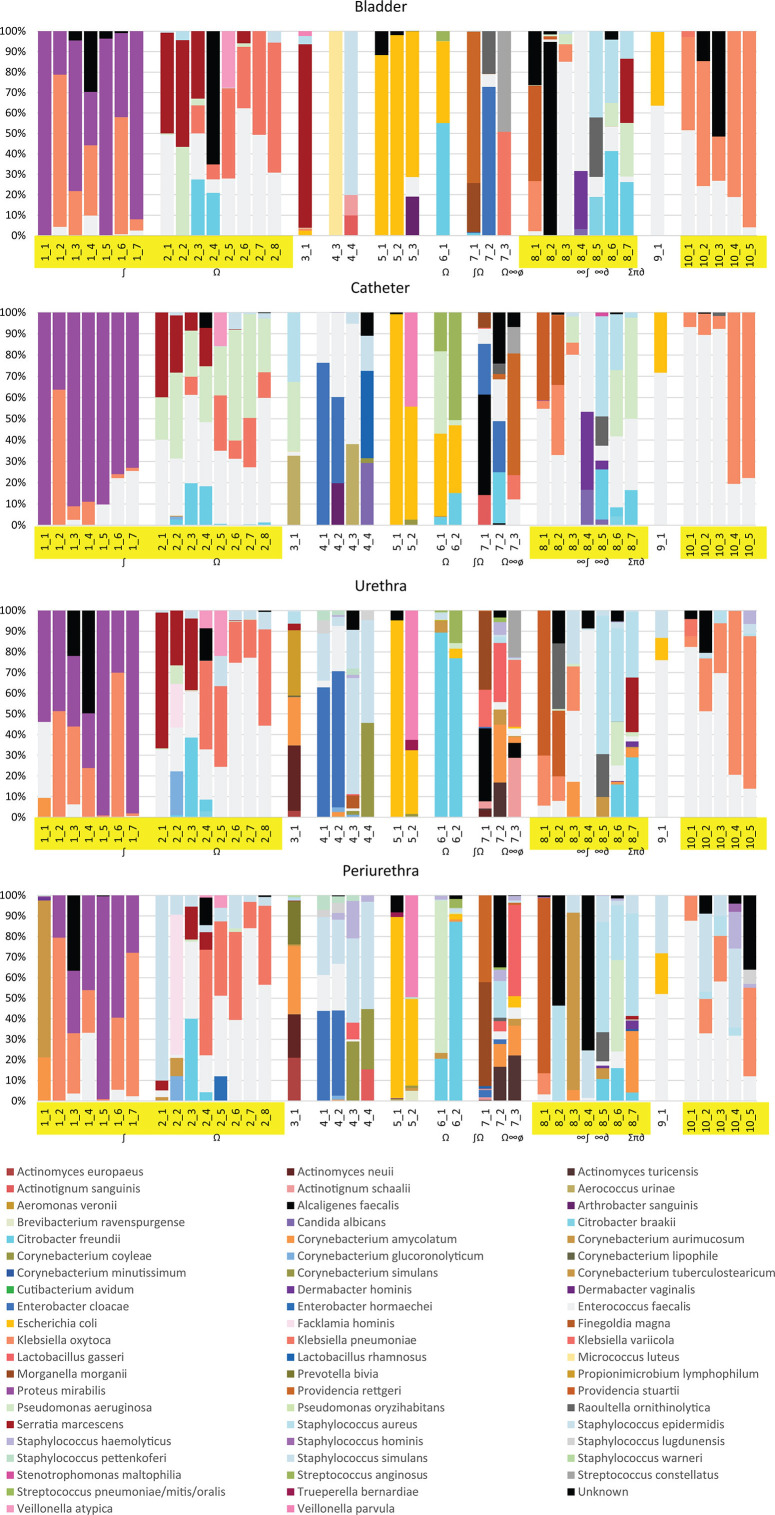
Microbiota composition profiles of each niche for all 10 participants at all time points. Top: Relative abundance of species identified using EQUC. Participants and time points denoted on x-axis with Participant_TimePoint. Highlighted participants are those with specimens collected ≥5 time points (participants 1, 2, 8, and 10). Symbols indicate antibiotic courses prescribed between collected time points. ∫ =trimethoprim–sulfamethoxazole; Ω = cephalexin; ∞ = ceftriaxone; ø = levofloxacin; ∂ = ciprofloxacin; ∑ = ampicillin; π = amoxicillin.Bottom: Legend, species level.

The relative abundance of species identified by EQUC for each participant and all four niches (bladder, catheter, urethra, and periurethra) is presented in Fig. 1. The urinary tract microbiota composition differed by individual participant, and this was observed at every time point and across all niches. The most predominant species observed in each participant were relatively consistent across all four niches. In participant 1, *Proteus mirabilis* was the most commonly observed species, followed by *Klebsiella oxytoca* and *Enterococcus faecalis*. In participant 2, the most predominant species observed were *E. faecalis*, *Klebsiella pneumoniae*, *Serratia marcescens*, *Citrobacter freundii*, and *Pseudomonas aeruginosa*. In participant 8, the most predominant species observed were *E. faecalis*, *Staphylococcus aureus*, *Providencia stuartii*, and *K. oxytoca*. In participant 10, the most predominant species was *E. faecalis*, followed by *K. oxytoca* and *S. epidermidis*. The complete list of species identified by EQUC is presented in Table S2.

When participants were pooled by niche, the average CFUs/mL isolated from each niche tended to be similar (mean ~ 2 x 10^5^). The most abundant species included commonly accepted uropathogens, such as members of the Gram-negative order Enterobacteriales (*Enterobacter cloacae, Providencia stuartii, Escherichia coli,* and *Klebsiella* species)*, Pseudomonas aeruginosa,* and the Gram-positive *Staphylococcus aureus*. They also included emerging opportunistic uropathogens, such as *Streptococcus anginosus* and *Alcaligenes faecalis* (Table 3).

**TABLE 3:** Total average CFU/mL, average CFUs/mL and five most abundant species isolated using EQUC by niche^*a*^

Niche	Total average CFU/mL and average CFUs/mL of all species identified using EQUC	Five most abundant species identified using EQUC (% of total CFUs/mL)
Bladder	6.99 × 10^6^/1.98 x 10^5^	*Enterobacter cloacae* (25.04) *Alcaligenes faecalis* (14.30) *Klebsiella oxytoca* (7.71) *Escherichia coli* (7.26) *Streptococcus constellatus* (5.96)
Catheter	6.42 × 10^6^/2.05 x 10^5^	*Enterobacter cloacae* (12.76) *Streptococcus anginosus* (12.35) *Providencia stuartii* (9.16) *Alcaligenes faecalis* (7.94) *Arthrobacter sanguinis* (7.79)
Urethra	1.04 × 10^7^/2.50 x 10^5^	*Morganella morganii* (10.29) *Facklamia hominis* (9.57) *Providencia stuartii* (6.40) *Klebsiella pneumoniae* (5.43) *Enterobacter cloacae* (5.18)
Periurethra	7.28 × 10^6^/1.63 x 10^5^	*Providencia stuartii* (15.21) *Facklamia hominis* (6.87) *Veillonella parvula* (6.87) *Enterobacter cloacae* (6.60) *Pseudomonas aeruginosa* (6.04)

^*a*^ All 10 participants included.

The relative abundance of genera identified by EQUC and by 16S rRNA gene sequencing for each participant and all four niches is presented in Fig. S1 and S2**,** respectively. Whereas EQUC identified 36 genera, 16S rRNA gene sequencing identified 120 genera. The 10 most abundant genera identified by EQUC matched the 10 most abundant genera identified by sequencing, with the exception of *Enterobacter* and *Citrobacter*, which were not identified by sequencing. The complete list of genera identified by 16S rRNA gene sequencing is presented in Table S3.

### Microbiota comparison

**Fig 2:**
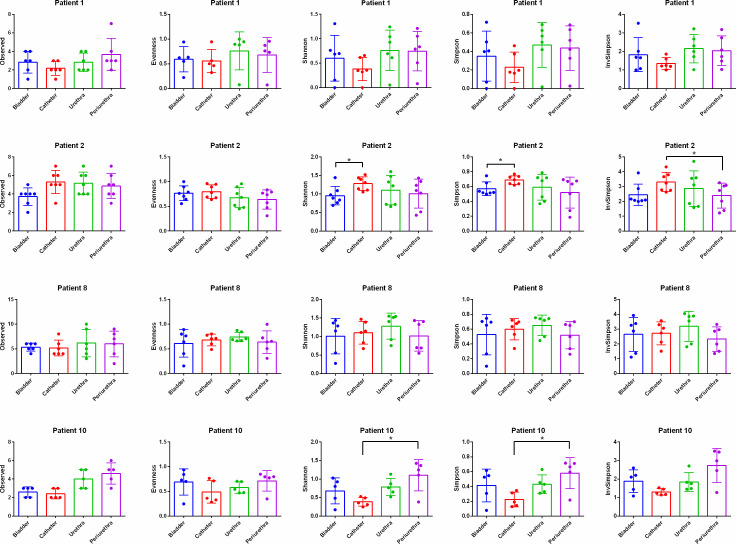
Alpha diversity measures (Observed, evenness, Shannon index, Simpson index, and Inverse Simpson index) of aggregated time points at each niche using EQUC. Participants with over three time points of specimen collection included. Tukey’s multiple comparison test compared alpha indices between niches with *P*-value at 0.05. 95% CI indicated with error bars. Significant differences between niches (*P* < 0.05) indicated with *.

**Fig 3:**
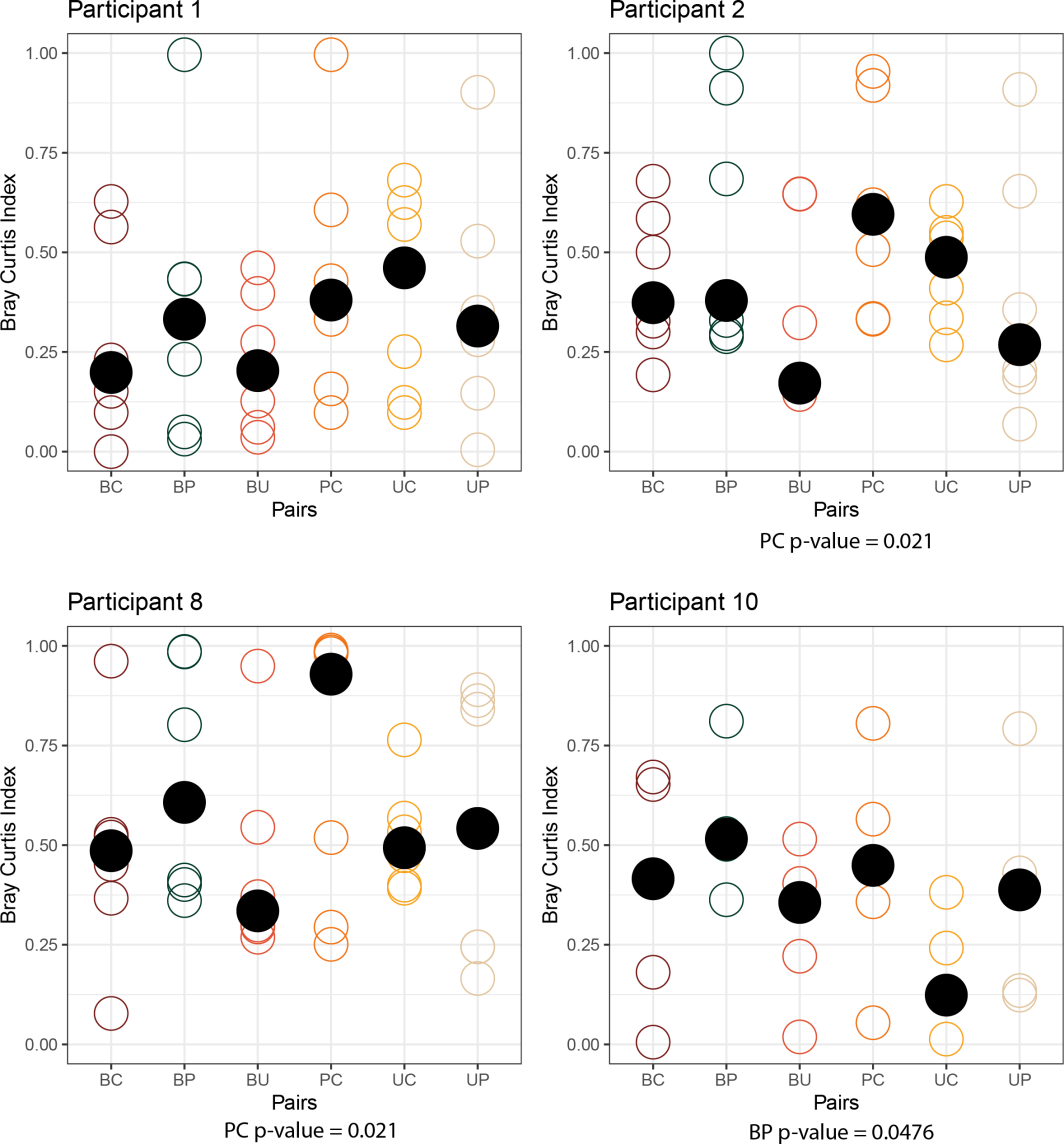
Microbiota diversity between niche pairs at the species level using EQUC for participants 1, 2, 8, and 10. Each open circle represents a Bray–Curtis dissimilarity value at one time point. Median dissimilarity score denoted by black dots. BC = bladder-catheter. BP = bladder-periurethra. BU = bladder-urethra. PC = periurethra-catheter. UC = urethra-catheter. UP = urethra-periurethra. Fisher’s exact test analyzed significance of Bray–Curtis dissimilarity scores with a 0.5 reference. *P*-values of significant pair dissimilarity values denoted under each panel where applicable.

Four participants had specimens collected from each niche at five or more time points; the within-specimen (alpha) diversity indices for these four participants based on EQUC and 16S rRNA gene sequencing data are shown in Fig. 2; Fig. S3, respectively. In general, there were very few significant differences between the four niches for any of the four participants and no consistent pattern observed by either method of detection. Another way to compare the microbiota of two niches is by calculating the Bray–Curtis Dissimilarity Index. Again, there were few significant differences and no obvious pattern (EQUC for participants 1, 2, 8, and 10: Fig. 3; EQUC for remaining participants: Fig. S4; 16S rRNA gene sequencing for all participants: Fig. S5).

**Fig 4:**
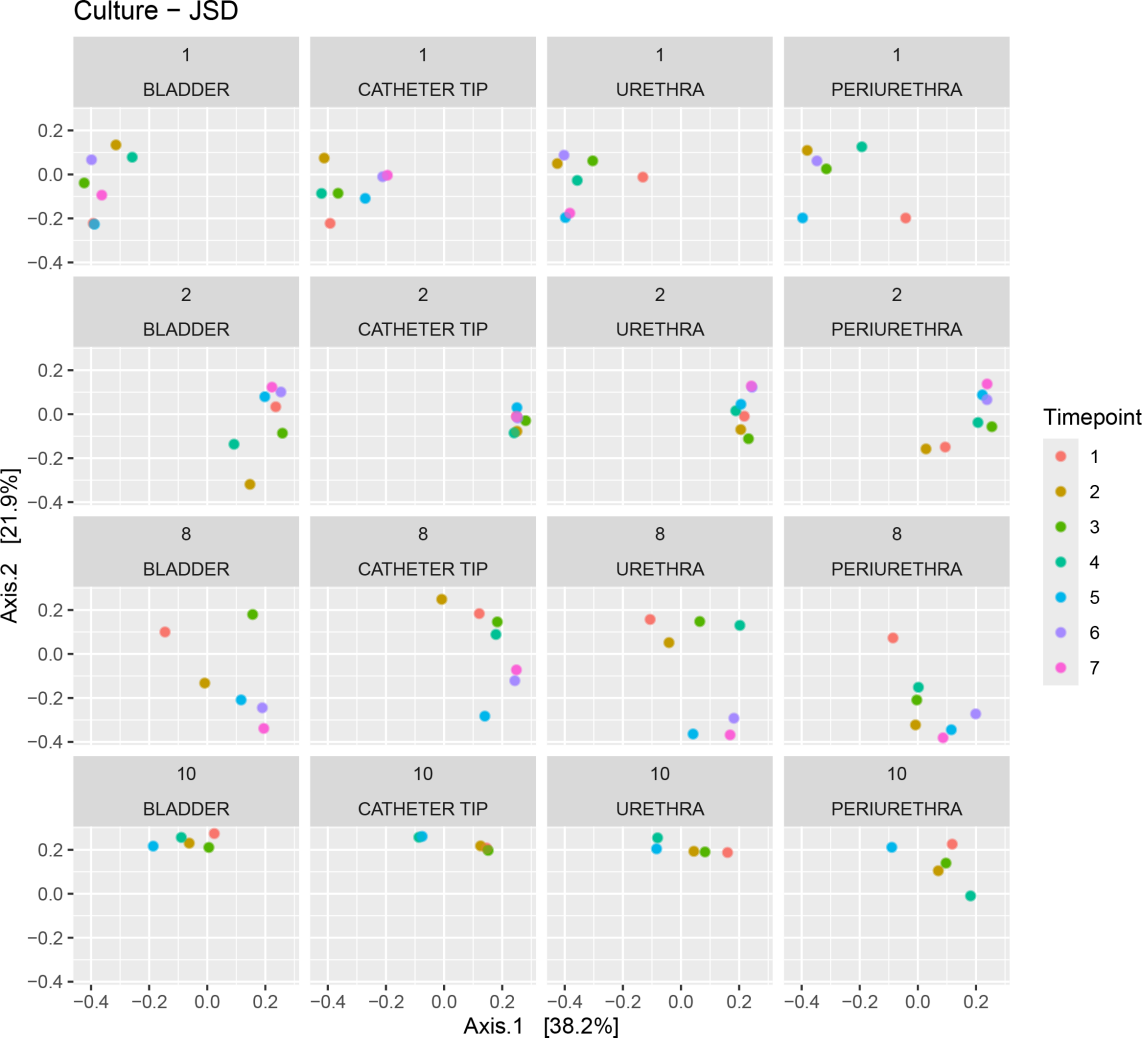
Microbiota composition comparison between all time points within each niche at the species level using EQUC. Jensen–Shannon Distance (JSD) matrix shown for participants 1, 2, 8, and 10. Each circle represents a JSD value of niche composition at corresponding time points compared to niche composition at all other time points.

Finally, we used Jensen–Shannon Distance (JSD) metrics to compare the microbiota of each niche for participants 1, 2, 8, and 10 over time (Fig. 4). In this analysis, each time point was compared to every other time point. Different patterns of temporal variation were observed for each participant. Whereas participants 2 and 10 had closely clustered JSD values, indicating less variation over time, the clusters for participants 1 and 8 were more widely distributed, indicating more variation in niche microbiotas over time. The JSD value matrix based on sequencing shows a similar pattern (Fig. S6).

## DISCUSSION

Despite precautions, CAUTI remains a prevalent disease that leads to increased morbidity, mortality, and antimicrobial drug resistance. Emerging evidence suggests an association between the state of the urobiome and UTI risk in adult females (38, 39). Studies examining links between the urobiome and CAUTI risk in patients with IUCs may also offer insights for appropriate CAUTI prevention and treatment. However, very few studies have characterized the urobiomes of those with IUCs (25–27), and even fewer have done so longitudinally (28, 29). And, to our knowledge, only Armbruster *et al*. has correlated the microbial characterization of urine clinically (29). However, the two longitudinal studies cited above operated under the assumption that bacteriuria is abnormal; the existence of the urinary microbiome was neither considered nor discussed.

In this exploratory longitudinal study, we compared the microbial composition of four niches—bladder, catheter, urethra, and periurethra—in ten geriatric male patients with chronic IUCs over several months. We then explored the alignment of the microbial composition data with clinical data regarding CAUTI diagnosis and treatment.

In all four niches across all participants, the most abundant microbes were “known” uropathogens, such as *E. cloacae*, *E. coli, K. oxytoca, P. mirabilis, E. faecalis,* and *S. aureus* but also the emerging uropathogen *S. anginosus*. Over the course of the study, however, the participants reported very few urinary signs and symptoms during the weekly follow-up calls, suggesting that the vast majority of the time, these microbes inhabited the urobiome of this patient population without associated symptoms. This is the defining property of an opportunistic pathogen: a microbe that can become pathogenic under certain circumstances but otherwise is beneficial or commensal. Thus, the presence of a microbe that can be pathogenic does not mean that it is currently causing symptoms (24). For example, *E. faecalis* has been shown to colonize IUCs by adhering to fibrinogen deposition secondary to host inflammatory reactions (40), but it may or may not cause urinary symptoms, depending on environmental circumstances. Our findings are consistent with those of two other known studies that characterized microbial communities in urine longitudinally; both studies found that most urine specimens from patients with chronic IUCs were polymicrobial and showed little variation in composition over time at the individual level. The most abundant species they detected was also *E. faecalis (*28, 29).

The utility of SUC for diagnosis and subsequent treatment guidance for UTI has been challenged by the discovery that urine is not sterile (11, 41). This is also true for CAUTI. As has been previously argued, the limitations of SUC result in the overreporting of certain microbes, especially *E. coli* and other fast-growing non-fastidious facultative anaerobes, and the underreporting of others, including *E. faecalis* and *S. anginosus* (11, 21, 23, 41). Our longitudinal clinical data, paired with urobiome data obtained by EQUC and 16S rRNA gene sequencing, suggest that CAUTI diagnoses made with SUC for three of the participants (participants 1, 2, and 8) may have been inappropriate. In all three cases, our longitudinal data show that the species identified by SUC was one of the most abundant in the participant’s bladder at time point(s) prior to the performance of SUC and, in two participants, the same species were also present at high abundance at time point(s) after SUC and antibiotic administration. For example, *P. mirabilis* was identified by EQUC in participant 1’s catheterized urine (bladder) specimens at all five time points prior to SUC performance at time point 6; in fact, *P. mirabilis* was the most abundant species identified the month prior to its identification on SUC, representing 96.4% of organisms in the urine specimen. SUC subsequently grew *P. mirabilis*, and trimethoprim–sulfamethoxazole was prescribed. However, *P. mirabilis* continued to be identified as the most predominant species present in the bladder at time point 7, accounting for 92% of the microbes in the specimen, despite treatment. The patient did not report any urinary signs or symptoms at any of the seven time points (Table 2). This raises the following questions: (1) would the clinician have made the same decision to diagnose CAUTI in these patients had they had access to longitudinal EQUC and/or sequencing data and (2) is treatment with antibiotics effective given the evidence that the assumed pathogen remained present at high numbers in the bladder after treatment? While the availability of longitudinal, better-than-SUC results might have changed decision-making concerning prescription of antimicrobials, the lack of symptoms (i.e., the failure of these participants to have met CAUTI diagnosis criteria) is concerning.

One prevailing theory of CAUTI etiology may be challenged by the existence of the urobiome: that the catheter acts as a conduit, specifically allowing extraluminally introduced microbes to ascend into the bladder along the external catheter surface and cause infection (13, 42). Now that we know microbes inhabit the bladder, the likelihood that uropathogens may not all be introduced extraluminally should be considered. Our data, however, are not inconsistent with the idea that an IUC can act as a conduit. There were very few significant differences between niche pairs in most participants, i.e., the microbiota of the four niches were very similar to one another. This finding is in contrast to the conclusions of a recent study of non-catheterized females with pelvic floor symptoms, in which the bladder microbiota were distinct from those of the urethra and periurethra (43). It also contradicts a recent study of non-catheterized older adult males with benign prostate hyperplasia, in which the bladder microbiota tended to differ from those of the urethra (44). Although our data showed that niche microbiotas were similar, we could not determine directionality—our data could not distinguish between ascension from the periurethra to the bladder from possible descension from the bladder to the periurethra.

The major strength of this study is the novel, longitudinal description of the microbiota in a male, geriatric, chronically catheterized population, assessed with both EQUC and 16S rRNA gene sequencing methods, microbial detection methods that are significant improvements to SUC (21, 23, 41). The small specimen size was a limitation, with only 10 participants in total and only four who completed the study with at least five time points of data processed. Another limitation was that diagnosis and treatment decisions of CAUTI were left to the provider instead of members of the study team. We relied solely on the patients’ responses to the questionnaire that our nurse coordinator utilized during their weekly calls to the patients when they were screened for signs and symptoms of UTI. On some occasions, the calls occurred after the patients had been started on antibiotics. Moreover, some patients were started on antibiotics by a provider outside of our health system and, thus, documentation was not available for us to review. Additionally, one major limitation was that all participants had chronic IUCs in place prior to the study start date, so no data describing the microbiota prior to catheterization could be obtained. This is one reason why we could not determine the directionality of microbial composition between niches. Our data support that sampling the bladder niche alone, via catheterized urine, may be sufficient for future studies.

This exploratory study described the urobiomes of geriatric male patients with chronic IUCs. The data show that longitudinal EQUC or 16S rRNA gene sequencing data could be useful to clinicians when diagnosing or treating possible CAUTI.

## Data Availability

Raw sequencing reads are publicly available in the NCBI SRA database (BioProject PRJNA1063747).

## SUPPLEMENTAL MATERIAL

Supplemental information spectrum.00941-24-s0001.docx Supplemental Information contains Table S1 and Figures S1-6.

Table S2 spectrum.00941-24-s0002.xlsx Average of sum of colony forming units per milliliter (CFUs/mL) isolated using EQUC at species level for each niche at each time point for all 10 participants.

Table S3 spectrum.00941-24-s0003.xlsx Read counts of 50 most abundant genera isolated using sequencing for each niche at each time point for all 10 participants.

ASM does not own the copyrights to Supplemental Material that may be linked to, or accessed through, an article. The authors have granted ASM a non-exclusive, world-wide license to publish the Supplemental Material files. Please contact the corresponding author directly for reuse.

## References

[1] Gould C. Catheter-associated urinary tract infection (CAUTI) Toolkit. CDC. Available from: https://www.cdc.gov/hai/pdfs/toolkits/cautitoolkit_3_10.pdf

[2] Urinary tract infection (catheter-associated urinary tract infection [CAUTI] and non-catheter-associated urinary tract infection [UTI]) events. 2023. National Healthcare Safety Network

[3] Chenoweth CE. 2021. Urinary tract infections: 2021 update. Infect Dis Clin North Am 35:857–870. doi:10.1016/j.idc.2021.08.003 34752223

[4] Fink R, Gilmartin H, Richard A, Capezuti E, Boltz M, Wald H. 2012. Indwelling urinary catheter management and catheter-associated urinary tract infection prevention practices in Nurses Improving Care for Healthsystem Elders hospitals. Am J Infect Control 40:715–720. doi:10.1016/j.ajic.2011.09.017 22297241

[5] Burton DC, Edwards JR, Srinivasan A, Fridkin SK, Gould CV. 2011. Trends in catheter-associated urinary tract infections in adult intensive care units-United States, 1990-2007. Infect Control Hosp Epidemiol 32:748–756. doi:10.1086/660872 21768757

[6] Lo E, Nicolle LE, Coffin SE, Gould C, Maragakis LL, Meddings J, Pegues DA, Pettis AM, Saint S, Yokoe DS. 2014. Strategies to prevent catheter-associated urinary tract infections in acute care hospitals: 2014 update. Infect Control Hosp Epidemiol 35:464–479. doi:10.1086/675718 24709715

[7] Flores-Mireles A, Hreha TN, Hunstad DA. 2019. Pathophysiology, treatment, and prevention of catheter-associated urinary tract infection. Top Spinal Cord Inj Rehabil 25:228–240. doi:10.1310/sci2503-228 31548790 PMC6743745

[8] Guideline for prevention of catheter-associated urinary tract infections. 2015. CDC. Available from: https://www.cdc.gov/infectioncontrol/guidelines/cauti/background.html 10.1016/s0196-6553(83)80012-1 6551151

[9] Murphy C, Cowan A, Moore K, Fader M. 2018. Managing long term indwelling urinary catheters. BMJ 363:k3711. doi:10.1136/bmj.k3711 30309871

[10] Finucane TE. 2017. “Urinary tract infection"-requiem for a heavyweight. J Am Geriatr Soc 65:1650–1655. doi:10.1111/jgs.14907 28542707

[11] Brubaker L, Chai TC, Horsley H, Khasriya R, Moreland RB, Wolfe AJ. 2023. Tarnished gold—the “standard” urine culture: reassessing the characteristics of a criterion standard for detecting urinary microbes. Front Urol 3. doi:10.3389/fruro.2023.1206046 PMC12327316 40778073

[12] Weiner-Lastinger LM, Abner S, Edwards JR, Kallen AJ, Karlsson M, Magill SS, Pollock D, See I, Soe MM, Walters MS, Dudeck MA. 2020. Antimicrobial-resistant pathogens associated with adult healthcare-associated infections: summary of data reported to the National Healthcare Safety Network, 2015-2017. Infect Control Hosp Epidemiol 41:1–18. doi:10.1017/ice.2019.296 31767041 PMC8276252

[13] Tambyah PA, Halvorson KT, Maki DG. 1999. A prospective study of pathogenesis of catheter-associated urinary tract infections. Mayo Clin Proc 74:131–136. doi:10.4065/74.2.131 10069349

[14] Fekete T. 2022. Catheter-associated urinary tract infection in adults. UpToDate

[15] Wolfe AJ, Toh E, Shibata N, Rong R, Kenton K, Fitzgerald M, Mueller ER, Schreckenberger P, Dong Q, Nelson DE, Brubaker L. 2012. Evidence of uncultivated bacteria in the adult female bladder. J Clin Microbiol 50:1376–1383. doi:10.1128/JCM.05852-11 22278835 PMC3318548

[16] Brubaker L, Nager CW, Richter HE, Visco A, Nygaard I, Barber MD, Schaffer J, Meikle S, Wallace D, Shibata N, Wolfe AJ. 2014. Urinary bacteria in adult women with urgency urinary incontinence. Int Urogynecol J 25:1179–1184. doi:10.1007/s00192-013-2325-2 24515544 PMC4128900

[17] Pearce MM, Zilliox MJ, Rosenfeld AB, Thomas-White KJ, Richter HE, Nager CW, Visco AG, Nygaard IE, Barber MD, Schaffer J, Moalli P, Sung VW, Smith AL, Rogers R, Nolen TL, Wallace D, Meikle SF, Gai X, Wolfe AJ, Brubaker L, Pelvic Floor Disorders Network. 2015. The female urinary microbiome in urgency urinary incontinence. Am J Obstet Gynecol 213:347. doi:10.1016/j.ajog.2015.07.009 PMC4556587 26210757

[18] Thomas-White KJ, Gao X, Lin H, Fok CS, Ghanayem K, Mueller ER, Dong Q, Brubaker L, Wolfe AJ. 2018. Urinary microbes and postoperative urinary tract infection risk in urogynecologic surgical patients. Int Urogynecol J 29:1797–1805. doi:10.1007/s00192-018-3767-3 30267143 PMC6527134

[19] Wolfe AJ, Brubaker L. 2019. Urobiome updates: advances in urinary microbiome research. Nat Rev Urol 16:73–74. doi:10.1038/s41585-018-0127-5 30510275 PMC6628711

[20] Whiteside SA, Razvi H, Dave S, Reid G, Burton JP. 2015. The microbiome of the urinary tract--a role beyond infection. Nat Rev Urol 12:81–90. doi:10.1038/nrurol.2014.361 25600098

[21] Price TK, Dune T, Hilt EE, Thomas-White KJ, Kliethermes S, Brincat C, Brubaker L, Wolfe AJ, Mueller ER, Schreckenberger PC. 2016. The clinical urine culture: enhanced techniques improve detection of clinically relevant microorganisms. J Clin Microbiol 54:1216–1222. doi:10.1128/JCM.00044-16 26962083 PMC4844725

[22] Deen NS, Ahmed A, Tasnim NT, Khan N. 2023. Clinical relevance of expanded quantitative urine culture in health and disease. Front Cell Infect Microbiol 13:1210161. doi:10.3389/fcimb.2023.1210161 37593764 PMC10428011

[23] Hochstedler BR, Burnett L, Price TK, Jung C, Wolfe AJ, Brubaker L. 2022. Urinary microbiota of women with recurrent urinary tract infection: collection and culture methods. Int Urogynecol J 33:563–570. doi:10.1007/s00192-021-04780-4 33852041 PMC8514570

[24] Moreland RB, Choi BI, Geaman W, Gonzalez C, Hochstedler-Kramer BR, John J, Kaindl J, Kesav N, Lamichhane J, Lucio L, Saxena M, Sharma A, Tinawi L, Vanek ME, Putonti C, Brubaker L, Wolfe AJ. 2023. Beyond the usual suspects: emerging uropathogens in the microbiome age. Front Urol 3. doi:10.3389/fruro.2023.1212590 PMC12327349 40778068

[25] Bossa L, Kline K, McDougald D, Lee BB, Rice SA. 2017. Urinary catheter-associated microbiota change in accordance with treatment and infection status. PLoS One 12:e0177633. doi:10.1371/journal.pone.0177633 28628622 PMC5476236

[26] Forster CS, Panchapakesan K, Stroud C, Banerjee P, Gordish-Dressman H, Hsieh MH. 2020. A cross-sectional analysis of the urine microbiome of children with neuropathic bladders. J Pediatr Urol 16:593. doi:10.1016/j.jpurol.2020.02.005 PMC7434660 32171668

[27] Lane G, Gracely A, Bassis C, Greiman SE, Romo PB, Clemens JQ, Gupta P, O’Dell D, Stoffel JT, Cameron AP. 2022. Distinguishing features of the urinary bacterial microbiome in patients with neurogenic lower urinary tract dysfunction. J Urol 207:627–634. doi:10.1097/JU.0000000000002274 34698526 PMC9197513

[28] Nye TM, Zou Z, Obernuefemann CLP, Pinkner JS, Lowry E, Kleinschmidt K, Bergeron K, Klim A, Dodson KW, Flores-Mireles AL, Walker JN, Wong DG, Desai A, Caparon MG, Hultgren SJ. 2024. Microbial co-occurrences on catheters from long-term catheterized patients. Nat Commun 15:61. doi:10.1038/s41467-023-44095-0 38168042 PMC10762172

[29] Armbruster CE, Brauer AL, Humby MS, Shao J, Chakraborty S. 2021. Prospective assessment of catheter-associated bacteriuria clinical presentation, epidemiology, and colonization dynamics in nursing home residents. JCI Insight 6:e144775. doi:10.1172/jci.insight.144775 34473649 PMC8525589

[30] Jung CE, Chopyk J, Shin JH, Lukacz ES, Brubaker L, Schwanemann LK, Knight R, Wolfe AJ, Pride DT. 2019. Benchmarking urine storage and collection conditions for evaluating the female urinary microbiome. Sci Rep 9:13409. doi:10.1038/s41598-019-49823-5 31527753 PMC6746804

[31] Schulz L, Hoffman RJ, Pothof J, Fox B. 2016. Top ten myths regarding the diagnosis and treatment of urinary tract infections. J Emerg Med 51:25–30. doi:10.1016/j.jemermed.2016.02.009 27066953

[32] Hilt EE, McKinley K, Pearce MM, Rosenfeld AB, Zilliox MJ, Mueller ER, Brubaker L, Gai X, Wolfe AJ, Schreckenberger PC. 2014. Urine is not sterile: use of enhanced urine culture techniques to detect resident bacterial flora in the adult female bladder. J Clin Microbiol 52:871–876. doi:10.1128/JCM.02876-13 24371246 PMC3957746

[33] Gao X, Lin H, Revanna K, Dong Q. 2017. A Bayesian taxonomic classification method for 16S rRNA gene sequences with improved species-level accuracy. BMC Bioinformatics 18:247. doi:10.1186/s12859-017-1670-4 28486927 PMC5424349

[34] Davis NM, Proctor DM, Holmes SP, Relman DA, Callahan BJ. 2018. Simple statistical identification and removal of contaminant sequences in marker-gene and metagenomics data. Microbiome 6:226. doi:10.1186/s40168-018-0605-2 30558668 PMC6298009

[35] McMurdie PJ, Holmes S. 2013. phyloseq: an R package for reproducible interactive analysis and graphics of microbiome census data. PLoS One 8:e61217. doi:10.1371/journal.pone.0061217 23630581 PMC3632530

[36] Dixon P. 2003. VEGAN, a package of R functions for community ecology. J Veg Sci 14:927–930. doi:10.1111/j.1654-1103.2003.tb02228.x

[37] Goslee SC, Urban DL. 2007. The ecodist package for dissimilarity-based analysis of ecological data. J Stat Softw 22:1–19. doi:10.18637/jss.v022.i07

[38] Brubaker L, Putonti C, Dong Q, Wolfe AJ. 2021. The human urobiome. Mamm Genome 32:232–238. doi:10.1007/s00335-021-09862-8 33651197

[39] Brubaker L, Gourdine J-PF, Siddiqui NY, Holland A, Halverson T, Limeria R, Pride D, Ackerman L, Forster CS, Jacobs KM, Thomas-White KJ, Putonti C, Dong Q, Weinstein M, Lukacz ES, Karstens L, Wolfe AJ. 2021. Forming consensus to advance urobiome research. mSystems 6:e0137120. doi:10.1128/mSystems.01371-20 34282932 PMC8409733

[40] Flores-Mireles AL, Walker JN, Bauman TM, Potretzke AM, Schreiber HL 4th, Park AM, Pinkner JS, Caparon MG, Hultgren SJ, Desai A. 2016. Fibrinogen release and deposition on urinary catheters placed during urological procedures. J Urol 196:416–421. doi:10.1016/j.juro.2016.01.100 26827873 PMC4965327

[41] Price TK, Hilt EE, Dune TJ, Mueller ER, Wolfe AJ, Brubaker L. 2018. Urine trouble: should we think differently about UTI? Int Urogynecol J 29:205–210. doi:10.1007/s00192-017-3528-8 29279968

[42] Klein RD, Hultgren SJ. 2020. Urinary tract infections: microbial pathogenesis, host-pathogen interactions and new treatment strategies. Nat Rev Microbiol 18:211–226. doi:10.1038/s41579-020-0324-0 32071440 PMC7942789

[43] Chen YB, Hochstedler B, Pham TT, Acevedo-Alvarez M, Mueller ER, Wolfe AJ. 2020. The urethral microbiota: a missing link in the female urinary microbiota. J Urol 204:303–309. doi:10.1097/JU.0000000000000910 32118507

[44] Bajic P, Van Kuiken ME, Burge BK, Kirshenbaum EJ, Joyce CJ, Wolfe AJ, Branch JD, Bresler L, Farooq AV. 2020. Male bladder microbiome relates to lower urinary tract symptoms. Eur Urol Focus 6:376–382. doi:10.1016/j.euf.2018.08.001 30143471

