# Supplementary material for: Characterizing the urobiome in geriatric males with chronic indwelling urinary catheters: an exploratory longitudinal study
Source: Microbiol Spectr. 2024 Oct 10;12(11):e00941-24. doi: 10.1128/spectrum.00941-24 (PMC11536997; doi:10.1128/spectrum.00941-24)
Supplement: Supplemental information — contains Table S1 and Figures S1-6. [file spectrum.00941-24-s0001.docx]

Characterizing the urobiome in geriatric males

with chronic indwelling urinary catheters:

an exploratory longitudinal study

Emma Stewart, Baylie R. Hochstedler-Kramer MS, Mark Khemmani MS, Nina M. Clark MD, Jorge P. Parada MD MPH, Ahmer Farooq DO, Chirag Doshi MD, Alan J. Wolfe PhD, Fritzie S. Albarillo MD

Supplemental Information

**Supplemental Table 1.**

| *Participant* | *Number of total visits during study* | *Number of visits during which specimen sets were collected* | *Number of specimen sets processed with EQUC* | *Number of specimen sets processed with 16S sequencing* |
| --- | --- | --- | --- | --- |
| *1 †▴* | 12 | 12 | 7 | 11 |
| *2 ▴* | 12 | 11 | 8 | 9 |
| *3* | 1 | 1 | 1 | 1 |
| *4 †* | 4 | 4 | 4 | 4 |
| *5* | 5 | 3 | 3 | 3 |
| *6 ▴* | 5 | 2 | 2 | 2 |
| *7 ▴* | 5 | 3 | 3 | 3 |
| *8 ▴* | 12 | 8 | 7 | 8 |
| *9* | 2 | 1 | 1 | 1 |
| *10 ▴* | 9 | 5 | 5 | 5 |

***Total number of visits, number of visits at which specimen sets were collected, and number of specimen sets processed by EQUC and 16S rRNA gene*** ***sequencing for each participant.*** *(*† *denotes patient deceased;*▴ *denotes patient diagnosed with CAUTI during study.)*

**Supplemental Figure 1.** *Microbiota composition profiles at genus level of each niche for all 10 participants at all time points. Relative abundance of genera identified using EQUC. Participants and time points denoted on x-axis with Participant_TimePoint.*

**Supplemental Figure 2.** *Microbiota composition profiles of each niche for all 10 participants at all time points. Relative abundance of top 50 genera identified using sequencing (matched to genera identified using EQUC). Participants and time points denoted on x-axis with Participant_TimePoint.*

**Supplemental Figure 3.** *Alpha diversity measures (Observed, evenness, Shannon index, Simpson index, Inverse Simpson index) of aggregated time points at each niche using sequencing. Participants with over 3 time points of specimen collection included. Tukey multiple comparison test compared alpha indices between niches with p-value at 0.05. 95% confidence interval indicated with error bars. Significant differences between niches indicated: p<0.05 = *, p<0.01 = **.*

**Supplemental Figure 4.** *Microbiota diversity between niche pairs at the species level using EQUC for participants at which specimens were collected at 4 time points or less (participants 3, 4, 5, 6, 7, and 9). Each open circle represents a Bray-Curtis dissimilarity value at one time point.* *Median dissimilarity score denoted by black dot. BC = bladder-catheter. BP = bladder-periurethra. BU = bladder-urethra. PC = periurethra-catheter. UC = urethra-catheter. UP = urethra-periurethra. Fisher’s exact test analyzed significance of Bray-Curtis dissimilarity scores with a 0.5 reference. No significant differences between paired dissimilarity values observed; p-values not shown.*

**Supplemental Figure 5.** *Microbiota diversity between niche pairs at the genus level using 16S sequencing for all participants. Each open circle represents a Bray-Curtis dissimilarity value at one time point.* *Median dissimilarity score denoted by black dot. BC = bladder-catheter. BP = bladder-periurethra. BU = bladder-urethra. PC = periurethra-catheter. UC = urethra-catheter. UP = urethra-periurethra. Fisher’s exact test analyzed significance of Bray-Curtis dissimilarity scores with a 0.5 reference. P-values of significant pair dissimilarity values denoted under each panel where applicable.*

**Supplemental Figure 6.** *Microbiota composition comparison between all time points within each niche at the genera level using sequencing. Jensen-Shannon Distance (JSD) matrix shown for participants 1, 2, 8, and 10. Each circle represents a JSD value of niche composition at corresponding time point compared to niche composition at all other time points.*
